# Genome survey and SSR analysis of *Apocynum venetum*

**DOI:** 10.1042/BSR20190146

**Published:** 2019-06-25

**Authors:** Guo-qi Li, Li-xiao Song, Chang-qing Jin, Miao Li, Shi-pei Gong, Ya-fang Wang

**Affiliations:** 1 Breeding Base for State Key Laboratory of Land Degradation and Ecological Restoration in Northwest China, Yinchuan 750021, Ningxia, P.R. China; 2 Key Laboratory for Restoration and Reconstruction of Degraded Ecosystem in Northwest China of Ministry of Education, Ningxia 750021, P.R. China; 3 Ningxia Academy of Agro-Forestry Science, Ningxia 750002, P.R. China

**Keywords:** Apocynum venetum, genome sequencing, GC content, heterozygosity ratio, K-mer analysis, SSR molecular marker

## Abstract

*Apocynum venetum* is an eco-economic plant that exhibits high stress resistance. In the present paper, we carried out a whole-genome survey of *A. venetum* in order to provide a foundation for its whole-genome sequencing. High-throughput sequencing technology (Illumina NovaSep) was first used to measure the genome size of *A. venetum*, and bioinformatics methods were employed for the evaluation of the genome size, heterozygosity ratio, repeated sequences, and GC content in order to provide a foundation for subsequent whole-genome sequencing. The sequencing analysis results indicated that the preliminary estimated genome size of *A. venetum* was 254.40 Mbp, and its heterozygosity ratio and percentage of repeated sequences were 0.63 and 40.87%, respectively, indicating that it has a complex genome. We used k-mer = 41 to carry out a preliminary assembly and obtained contig N50, which was 3841 bp with a total length of 223949699 bp. We carried out further assembly to obtain scaffold N50, which was 6196 bp with a total length of 227322054 bp. We performed simple sequence repeat (SSR) molecular marker prediction based on the *A. venetum* genome data and identified a total of 101918 SSRs. The differences between the different types of nucleotide repeats were large, with mononucleotide repeats being most numerous and hexanucleotide repeats being least numerous. We recommend the use of the ‘2+3’ (Illumina+PacBio) sequencing combination to supplement the Hi-C technique and resequencing technique in future whole-genome research in *A. venetum*.

## Introduction

*Apocynum venetum* (Apocynaceae), also known as sword-leaf dogbane, is a perennial grass that is a valuable wild plant resource in China. It is also known as ‘the king of wildlife fibers’ due to the excellent quality of its fiber products. It was first discovered in the Luopu plains in Xinjiang province in the 1950s and was named ‘Luobu hemp’ [1]. It is an eco-economic plant with high stress resistance and is widely distributed in saline and alkaline deserts, desert boundaries, river banks, alluvial plains, lakes, and the Gobi Desert in China at latitudes of 35°–45°N [2]. Its leaves can be used to brew tea; its stems can be made into fibers; and it is also a source of honey. The roots, stems, and flowers of *A. venetum* are used in medicinal preparations. In 1977, *A. venetum* was listed in the *Pharmacopoeia of the People’s Republic of China* as a primary treatment for hypertension and hyperlipidemia, and is particularly suitable for treating constipation, obesity, and heart palpitations in middle-aged and elderly people. As a plant with important economic value, research on *A. venetum* has attracted widespread attention in recent years. Relevant studies have mainly focused on the physiological characteristics [3,4], pollination characteristics [5], medicinal components [6], germplasm resources [7], tissue culture [8,9], and *chalcone synthase* (*CHS*) cloning [10] of *A. venetum*, while very few studies have assessed the genetics of *A. venetum*. There are only 72 sequences for *A. venetum* available on the NCBI database (as of 21 July 2018). Limited gene sequence resources have restricted any in-depth research into the molecular biology of *A. venetum*, and thus the whole-genome sequencing of this economically valuable plant is warranted.

All life phenomena in organisms are associated with genes. The whole-genome sequencing of *A. venetum* is thus required to elucidate its high stress resistance and the characteristics associated with its high economic value. Omics technologies are constantly developing, particularly in genomics. Third-generation technologies have matured and are presently widely used, providing strong technical support for genome sequencing. In 2000, the whole-genome sequencing of a higher plant, *Arabidopsis thaliana*, was completed [11], which provided a foundation for whole-genome sequencing research in plants. Subsequently, whole-genome sequences from rice (*Oryza sativa*) [12], sorghum (*Sorghum bicolor*) [13], maize (*Zea mays*) [14] and many other plants have been published. This has provided a technical foundation for the genome sequencing of other plants. In order to fully mine molecular biology data from *A. venetum*, analyze crucial genes in the synthesis of *A. venetum* fibers, determine stress-related functional genes, and understand the nature of its stress resistance, we have sequenced and analyzed the genome of *A. venetum* in the present study. This will not only have significance for genomics and evolutionary research, but will also provide a foundation for the exploitation of the economic value of *A. venetum*. Before carrying out large-scale whole-genome deep sequencing in plants, it is necessary to first conduct low-coverage genome analysis and simple sequence repeat (SSR) molecular marker analysis in order to understand the compositional characteristics and patterns of the entire genome. K-mer analysis of low-depth sequencing data based on fragment libraries can be used to effectively assess genome size, GC content, heterozygosity ratio, and repeated sequences, and enables us to comprehensively evaluate the genome characteristics. This provides a basis for subsequent *de novo* sequencing and whole-genome assembly studies in *A. venetum*.

## Materials and methods

### Experimental materials

The *A. venetum* roots were collected from the *A. venetum* experimental base in Yinchuan City, Ningxia Province, at the end of March 2018 and were planted in pots. In early May, healthy plants exhibiting good growth were selected, and young leaves and stems at the apex were collected, snap-frozen in liquid nitrogen, and stored at −80°C until subsequent experiments.

### Experimental methods

#### Sample extraction and measurement

The modified cetyltrimethylammonium bromide (CTAB) method was used to extract *A. venetum* genomic DNA [15]. UV spectrophotometry was used to measure the concentration of the template (the ratio of absorbance at 260 and 280 nm was used to determine the purity and extraction results of the DNA). Agarose gel electrophoresis was used to determine the integrity of the template.

#### Sequencing data generation and quality control

The *A. venetum* DNA samples deemed to be of suitable quality were randomly sheared into 350-bp fragments using an ultrasonicator (Covaris Inc.). Electrophoresis was used to recover the DNA fragments of required lengths before end-repair, following which poly A-tail and sequencing adapters were added. The obtained fragments were purified before PCR amplification for library preparation. The Illumina NovaSep platform was used for high-throughput paired-end sequencing of the constructed libraries. In order to ensure the quality of the analysis, we filtered reads that would interfere with subsequent information, reads with adapters, reads with an N (unable to determine base information) ratio greater than 10%, and low-quality reads from the raw reads to obtain clean reads. The entire genome sequencing was carried out by Novogene Co. Ltd. (Beijing, China).

#### 17-mer analysis and prediction of genome size, heterozygosity ratio, and repeated sequences

K-mer analysis was used to predict the genome size, heterozygosity ratio, and repeated sequences of the clean reads before genome assembly. A K-value of 17 was used for the prediction, analysis, and iterative selection of 17-bp base sequences from the clean reads. We assumed that the K-mer depth frequency distribution followed a Poisson distribution and that all K-mers that were obtained base-by-base from the reads could cover the entire genome. Following this, we tallied the K-mer frequency distribution and calculated the K-mer depth distribution curve and depth product curve. The estimated K-mer depth value was obtained from the curve and used to estimate genome size [16,17]. The tailing phenomenon of the K-mer depth distribution curve was used to estimate the repeated sequences in the genome. A heterozygous genome includes two types: heterozygous K-mer and homozygous K-mer. Assuming that each heterozygous site is covered by 2×K K-mers, the expected depth of the heterozygous K-mer is ^1^/_2_. Therefore, the number of heterozygous sites can be estimated using ^1^/_2_ (percentage of heterozygous K-mer types) and *n*_Kspecies_ (total number of all K-mer types). The heterozygosity ratio was calculated using (eqn 1). The proportion of repeated sequences can be obtained by calculating the proportion of the number of K-mers greater than 1.8-fold of the homozygous peak depth.

(1) $$\varphi \;=\;\frac{a1/2\times n\text{Kspecies}/(2\times K)}{n\text{Kspecies}-a\text{1/2}\times n\text{Kspecies}/2}\;=\;\frac{a1/2}{K(2-a1/2)}$$

where a_1/2_ is the percentage of heterozygous K-mer types and *n*_Kspecies_ is the total number of all K-mer types.

#### Preliminary genome assembly

SOAPdenovo2 software was used to carry out preliminary genome assembly [18]. First, low frequency K-mers were corrected before K-mer = 41 was used to cut the corrected fragment library reads into even smaller sequence fragments. The overlap between these reads was used to construct a de Bruijn graph. A simplified de Bruijn graph was obtained from selection, simplification, and merging, and the sequences at every bifurcation locus were truncated to obtain the initial contigs. The reads obtained from sequencing all the libraries were aligned to the initially obtained contigs. The connectivity relationship between the reads and the information of the inserted fragment size were used to further assemble the contig into a scaffold and obtain the primary genomic sequence containing Ns. Following this, the filtered reads were aligned to this assembled sequence using SOAP to obtain the base depth. A window size of 10 kb was used for non-repetitive advancement in the sequence and calculation of the mean depth and GC content of every window to generate a GC depth plot. The graph could be used to examine whether there was significant GC bias or bacterial contamination in the sequences. Further, the stratification of GC clusters could be used to determine the heterozygosity ratio and percentage of repeated sequences of the genome [19].

#### Analysis of SSR molecular markers

MicroSatellite identification tool (MISA) software (http://pgrc.ipk-gatersleben.de/misa/misa.html), which was written using Perl, was used for the analysis of SSR molecular markers in the genome, to identify all microsatellite repeat units in the genome sequence, and to calculate the location, length, quantity, start sites, and end site of the SSRs in the scaffold. The parameters were set prior to MISA operation as follows: number of mononucleotide repeats ≥ 10, number of dinucleotide repeats ≥ 6, and number of trinucleotide, tetranucleotide, pentanucleotide, and hexanucleotide repeats ≥ 5.

## Results and discussion

### Sequencing data statistics and quality evaluation of *A. venetum*

**Figure 1:**
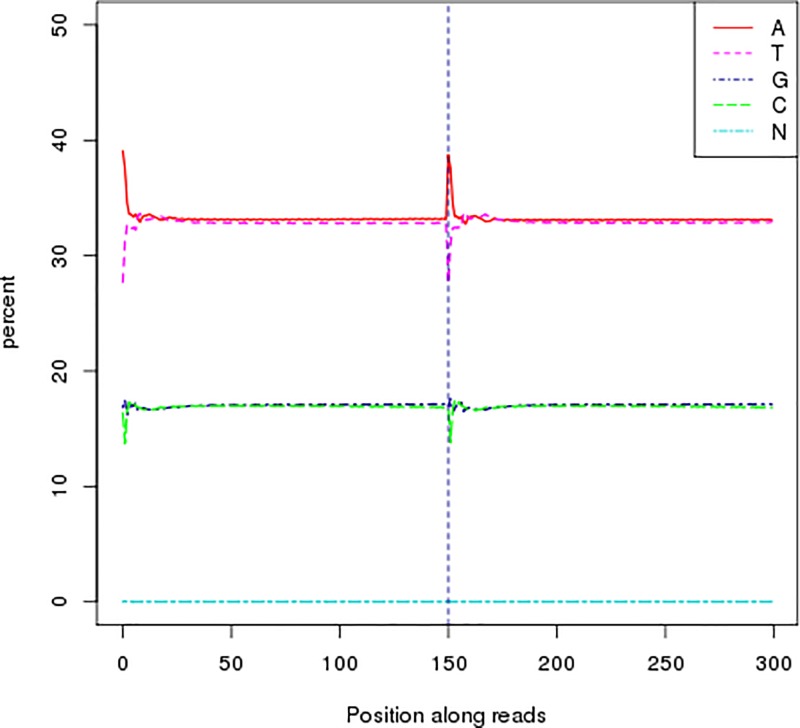
Distribution figure of GC content The left half of the dotted line in this figure is the read-1 GC content distribution, and the right half is the read-2 GC content distribution, different colors represent different base types, which is used to detect whether AT, GC separation is present.

The Illumina NovaSep platform was used for high-throughput paired-end sequencing to obtain 36.62 Gb of *A. venetum* raw bases. After filtering low-quality data, we obtained 36.61 Gb of clean bases, which accounted for 99.96% of the raw bases. In second-generation sequencing, a corresponding quality value will be obtained by sequencing every base. This quality value is an important marker for measuring sequencing accuracy. The higher the quality value (Q), the lower the probability that the base was incorrectly sequenced. The Illumina platform specifies that the Q20 and Q30 should be at least 90 and 85%, respectively. The sequencing quality evaluation showed that Q20 and Q30 were 96.09 and 89.95%, respectively (Table 1). This indicates that the high-throughput sequencing of *A. venetum* was highly accurate. Figure 1 shows the proportion of single bases, which is used to detect whether AT and GC separation is present. It can be seen that the content of A and G and C and T are close and the N content is almost zero. The results demonstrated that the sequencing quality was good.

**Table 1:** Data statistics

Lib ID	Raw base (bp)	Clean base (bp)	Effective rate (%)	Error rate (%)	Q20 (%)	Q30 (%)
NDES00224	36620525700	36605877489	99.96	0.04	96.09	89.95

Abbreviations: Q20, percentage of bases with quality value ≥ 20; Q30, percentage of bases with quality value ≥ 30.

### Results of 17-mer analysis and prediction of genome size, heterozygosity ratio, and percentage of repeated sequences

**Figure 2:**
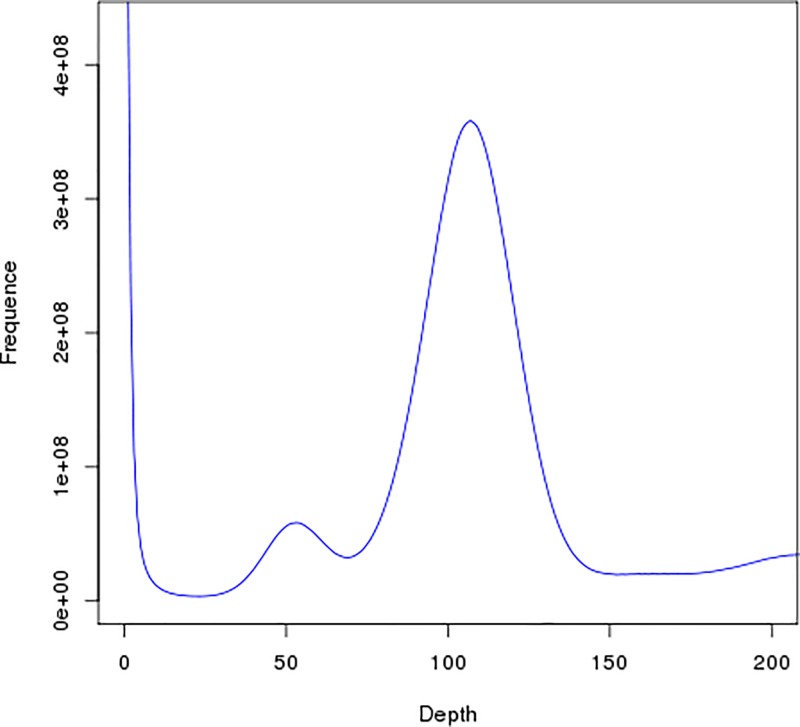
Distribution curve of K-mer It is an analysis of the genome size prediction of *Apocynum*, which determines the expected depth of K-mer from the position of the main peak.

We used 36.61 Gb of valid *A. venetum* genome data for K-mer analysis using a K-value of 17 and obtained a frequency distribution graph (Figure 2). The *x*-coordinates represent the K-mer depth and the *y*-coordinates represent the number of K-mers for the corresponding depth. From Figure 2, we can see that a depth = 106 is near the main peak, i.e. the expected depth of K-mers. SOAPdenovo software calculated the total number of K-mers as 27.48 Gb. A formula (genome size = K-mer number/K-mer depth) estimated the genome size to be approximately 259.25 Mbp. After excluding the error effects due to erroneous K-mers, the corrected genome size was found to be 254.40 Mbp. (eqn 1) was used for calculating the proportion of heterozygous sites in the sequence, obtaining a gene heterozygosity ratio of 0.63%. By calculating the percentage of 1.8-times the number of K-mers after the main peak over the total number of K-mers, we obtained a percentage of repeated sequences of 40.87%.

### Preliminary genome assembly results for *A. venetum*

**Figure 3:**
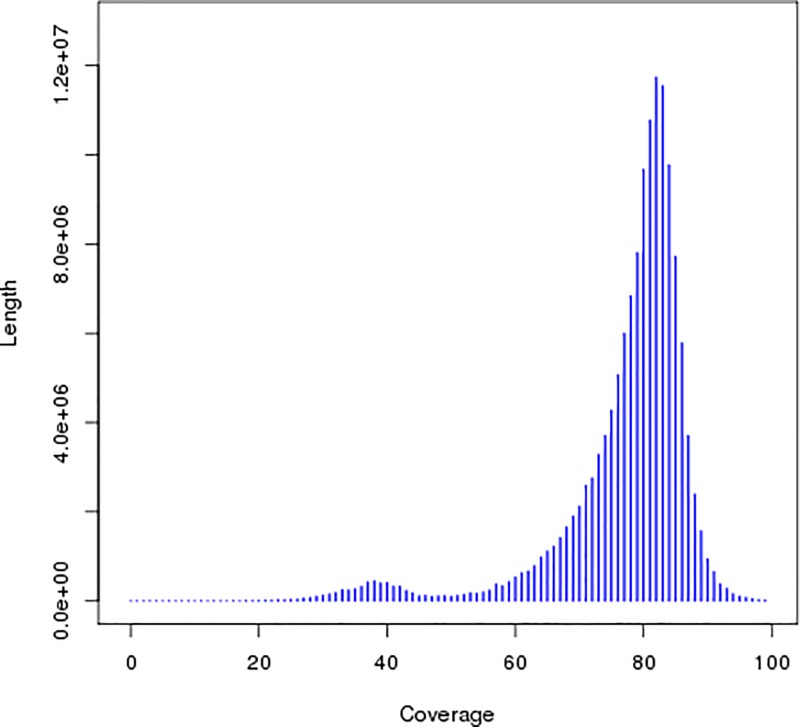
Distribution figure of contig coverage depth and length In the figure, the peak with the most distribution is the main peak, the heterozygosity of the genome was judged according to the peak of 1/2 position before the main peak.

**Figure 4:**
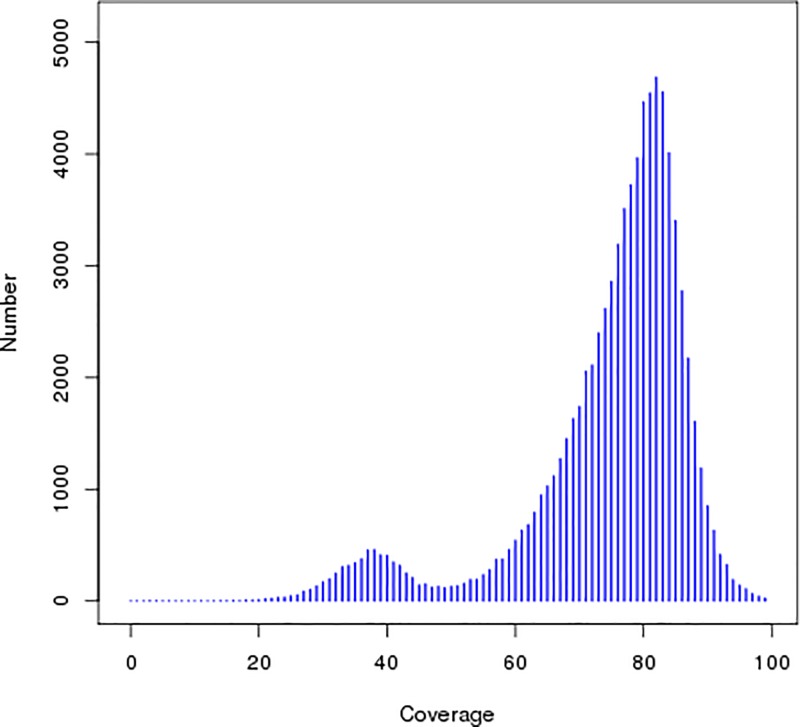
Distribution figure of contig coverage depth and number In the figure, the peak with the most distribution is the main peak, the heterozygosity of the genome was judged according to the peak of 1/2 position before the main peak.

The 36.61 Gb of clean bases was used for preliminary genome assembly, and a K-mer value of 41 was selected to construct the contig and scaffold, obtaining optimal assembly results. The results are shown in Table 2. We obtained a total of 282245 contigs with a total length of 223949699 bp, and the longest assembled sequence had a length of 134265 bp. The length of the N50 contig was 3841 bp. We obtained 239333 scaffolds after further assembly with a total length of 227322054 bp, and the longest sequence assembled was 191270 bp. The length of the N50 scaffold was 6502 bp. The results from Figures 3 and 4 showed significant peaks. It can be determined that the peak value at approximately 82x is a homozygous peak. The peak that is located around half of the *x-*coordinates in front of the homozygous peak is the heterozygous peak. Therefore, the genome of *A. venetum* is heterozygous and complex.

**Table 2:** Statistics of the assembled genome sequences in *A. venetum*

Item	Contig		Scaffold	
	Length (bp)	Number	Length (bp)	Number
N50	3841	10027	6196	6502
N60	2016	18198	3091	11744
N70	1074	33671	1468	22655
N80	562	62797	696	45616
N90	255	122424	300	96392
Total length (bp)	223949699		227322054	
Total number	282245		239333	
Max length (bp)	134265		191270	
GC content (%)	32.91			

### GC content and distribution status

**Figure 5:**
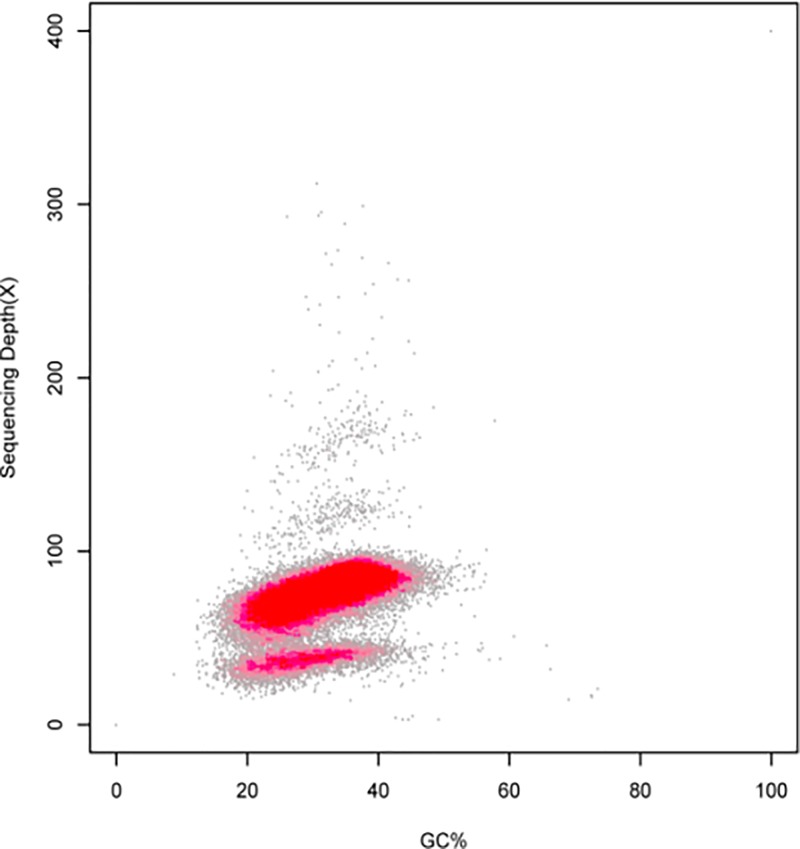
Distribution figure of GC_depth

GC_depth analysis (Figure 5) indicated that the GC content of almost all windows was 20–60%, and the sequencing depth was greater than 20-fold. The *A. venetum* samples did not show any apparent abnormalities, and there was no obvious GC bias. The *A. venetum* GC depth distribution was divided into two layers, and low depth was found in one region. The sequences of the low-depth distribution were extracted and Blast software was used to align these sequences to the NCBI Nucleotide (Nt) database. The results showed that these samples do not contain exogenous contamination. The GC clusters were divided into two obvious layers, which may be due to the heterozygosity. This is because heterozygosity causes the two homologous chromosomes at the heterozygous site to assemble into one or two strands. Additionally, the read products of these sites are half of the entire genome product. This causes a lower layer to appear in the GC content graph.

### Whole-genome SSR sequencing analysis of *A. venetum*

Using the MISA script, a total of 101918 SSRs in the *A. venetum* genome and the repeat units were used for the classification and calculation of nucleotide repeats. There were 65220 mononucleotide repeats, accounting for 63.99% of the total; 29276 dinucleotide repeats, accounting for 28.73% of the total; and 6632 trinucleotide repeats, accounting for 6.51% of the total. Tetranucleotide, pentanucleotide, and hexanucleotide repeats accounted for 0.52, 0.16, and 0.10% of the total, respectively. Therefore, the mononucleotide repeat was the main form in the *A. venetum* genome, while the proportion of hexanucleotide repeats was the lowest. Among the mononucleotide SSR repeats, A/T repeats were the most common. Among the dinucleotide SSR repeats, most repeats were AT/TA, accounting for 20.27% of the total, while CG/GC only accounted for 0.02% of the total (Table 3).

**Table 3:** Type and proportion of SSR

SSR repeat type	Number	Proportion (%)	SSR repeat type		Number	Proportion (%)
Mononucleotide						
A/T	59078	57.966	Tetranucleotide	AAAT/ATTT	248	0.243
C/G	6142	6.026		AACC/GGTT	1	0.001
Dinucleotide						
AC/GT	3050	2.993		AACT/AGTT	5	0.005
AG/CT	5552	5.448		AAGG/CCTT	4	0.004
AT/TA	20655	20.266		AATC/ATTG	6	0.006
CG/GC	19	0.019		AATG/ATTC	5	0.005
Trinucleotide						
AAC/GTT	120	0.118		AATT/AATT	37	0.036
AAG/CTT	1390	1.364		ACAG/CTGT	2	0.002
AAT/ATT	4020	3.944		ACAT/ATGT	42	0.041
ACC/GGT	215	0.211		ACCC/GGGT	4	0.004
ACG/CGT	27	0.026		ACTC/AGTG	2	0.002
ACT/AGT	135	0.132		AGAT/ATCT	25	0.025
AGC/CTG	124	0.122		AGCC/CTGG	1	0.001
AGG/CCT	166	0.163		AGGC/CCTG	1	0.001
ATC/ATG	395	0.388		AGGG/CCCT	10	0.010
CCG/CGG	40	0.039		ATCC/ATGG	4	0.004
Tetranucleotide						
AAAC/GTTT	18	0.018		ATGC/ATGC	2	0.002
AAAG/CTTT	112	0.110				

## Discussion

### Prediction of genome size

Whole-genome sequencing is a modern tool that enables the examination of the genetic code of plants. The whole-genome sequencing and construction of genome-wide maps in plants has promoted modern life science research. Genome size refers to the DNA content of all biological haploids and is usually described using the C-value. This value is a constant for every species and shows species-specific characteristics [20]. The genome can reflect all the genetic information of a biological species, and increasing studies have found that genome size is related to different biological parameters, such as stress resistance and economic characteristics. Increasingly, studies have found that genome size is related to different biological parameters, such as cell cycle and cell size, and genome size plays an important role in plant evolution and adaptation [21]. Methods for studying genome size range from renaturation kinetics [22], to pulsed-field gel electrophoresis [23], to flow cytometry [24,25], to modern high-throughput sequencing and K-mer estimation [26]. These methods are becoming increasingly convenient and the results are becoming more accurate. K-mer estimation of genome characteristics can greatly assist in exploring the genomes of unknown species. This method was successfully applied in the prediction of genome sizes for camphor tree (*Cinnamomum camphora*) [27], monk fruit (*Siraitia grosvenorii*) [19], Chinese tulip tree (*Liriodendron chinense*) [28], *Ammopiptanthus mongolicus* [29], and many other species. There is great variation in genome sizes among different species. Among angiosperm plants, the species with the smallest genome is *Genlisea tuberosa* from the family Lentibulariaceae, with a genome size of 61 Mbp [30]; while the species with the largest genome is the canopy plant (*Paris japonica*) from the family Melanthiaceae, with a genome size of 150 Gb [31]. The difference in genome size between these two species is approximately 2400-times. In the present study, the genome size of *A. venetum* was determined to be 254.40 Mbp (Table 1), which is close to the genome size of *Oropetium thomaeum* (245 Mbp) and *Kalanchoe fedtschenkoi* (260 Mbp)*.* A comparison of the published genome sizes of plants suggests that the *A. venetum* genome is relatively small, indicating that future genome assembly and annotation should be relatively simple. We have completed the genome survey of *A. cannabinum* and found its genome to be 239.02 Mbp in size, which is smaller than that of *A. venetum* [32]*.*

The genome survey of *A. venetum* has provided a solid foundation for its whole-genome sequencing, which will greatly accelerate and promote the exploration of this eco-economic plant, particularly its medicinal value. As a traditional medicine of China, *A. venetum* has long been used to calm the nerves, and promote diuresis, and the tea of roasted *A. venetum* leaves has been commercialized as a sedative and anti-aging supplement. Modern medical and pharmacological studies have indicated that *A. venetum* has broad pharmacological activities that include antihypertensive, cardiotonic, hepatoprotective, antioxidant, lipid-lowering, antidepressant, and anxiolytic effects. In additional, as an ethnopharmacological herb in Lop Nor region of China, *A. venetum* leaves have even been added to tobacco to detoxify nicotine [33]. One study verified that *A. venetum* leaf extract inhibits aortic contraction via its superoxide anion scavenging properties and nitric oxide releasing effect, which may account for its use as an antihypertensive treatment in traditional folk medicine [34]. Furthermore, an aqueous extract of *A. venetum* leaves could inhibit sodium channels, thus affecting neurotransmission and modulating neuronal ion channels, and thus may exert neuropharmacological effects [35].

### Genome GC content, heterozygosity ratio, and percentage of repeated sequences in *A. venetum*

The GC content of the *A. venetum* genome is 32.91% (Table 2), which is similar to *A****.*** *mongolicus* (36.51%) and *A. thaliana* (35.97%). It is lower than that of rice, maize, and sorghum, which have a GC content of more than 40%, but is higher than that of apples (*Malus pumila*) and alfalfa (*Medicago sativa*), which have a GC content of less than 30%. Shangguan et al. [36] summarized most of the available data on plant genomes and found that their GC contents mostly range within 30–47%. Another study also showed that excessively high (>65%) or excessively low (<25%) GC contents will result in errors in high-throughput sequencing and affect the accuracy of spliced data [37]. The heterozygosity ratio and percentage of repeated sequences in the sequencing data have important significance for guiding the assembly and splicing of genomes. In the present study, we calculated the heterozygosity ratio of *A. venetum* to be 0.63%. In the contig distribution graph (Figures 3 and 4), the peak located halfway in front of the main peak is the heterozygous peak. This also proved the existence of heterozygosity in the *A. venetum* genome. The size of the heterozygosity ratio can usually be used to divide genomes into low heterozygosity (0.5% ≤ heterozygosity ratio <0.8%), high heterozygosity (heterozygosity ratio ≥0.8%), highly repetitive genome (percentage of repeated sequences ≥50%), and low repetitive genomes (percentage of repeated sequences <50%) [27]. This preliminary analysis determined the *A. venetum* genome to have low heterozygosity. A K-mer distribution curve (Figure 2) was calculated, obtaining a repeated sequence percentage of 40.87%. Thus, the genome of *A. venetum* is complex with low heterozygosity and low repeated sequences. Repeated sequences are one of the major factors that control the recombination and regulation of structural genes and are also important components of non-coding regions. Repeated sequences exist in a state of dynamic variation, and gene expression may be precisely regulated by repeated sequences. Currently, there are three hypotheses to explain the existence of repeated sequences, namely the mutation-drift model, adaptive theory, and transposition mechanisms [21]. However, there is still a lack of understanding of the functions of repeated sequences in *A. venetum*, and further investigation is required.

### Whole-genome SSR marker characteristics of *A. venetum*

Molecular markers are an ideal form of genetic marker. In addition to facilitating detection, multiple allele polymorphism, and codominant inheritance, molecular markers also possess advantages that are not found in rapid fragment length polymorphism (RFLP) and amplified fragment length polymorphism (AFLP) markers. We found that mononucleotides were the most common SSR loci, with A/T ratios greater than the G/C ratio, accounting for 57.97% of the total number of repeat units. Among the dinucleotide repeats, AT/TA repeats were highest, accounting for 20.27% of the total number of repeat units, while CG/GC repeats were lowest, accounting for only 0.02% of the total. This may be due to methylation of cytosine into thymidine, resulting in a larger difference between these two nucleotide repeats [38]. Among the trinucleotides, the levels of AAT/TTA were the highest. This is similar to the distribution of trinucleotide sequences in *A. mongolicus* and grapes [39]. Statistical analysis of the differences in the quantity and types of SSRs in *A. venetum* and an initial exploration of the genome data have provided a foundation for the further construction of high-density genetic maps and the study of the regulatory mechanisms of *A. venetum* under stress conditions. This also provides a good reference for future genome and molecular marker research.

## Conclusion

This is the first study to measure the size of the entire *A. venetum* genome and preliminarily assess the corresponding parameters. The size of the *A. venetum* genome was estimated to be 259.25 Mbp, which was corrected to 254.40 Mbp. The heterozygosity ratio was 0.63% and the percentage of repeated sequences was 40.87%. We used K-mer = 41 to carry out a preliminary assembly and determined contig N50 to be 3841 bp, with a total length of 223949699 bp, while scaffold N50 was determined to be 6196 bp, with a total length of 227322054 bp. A total of 101918 SSRs were identified from the *A. venetum* genome data. There was great variation between the different types of nucleotide repeats, of which mononucleotides were the most abundant and hexanucleotides were the least abundant. Based on an analysis of various markers, we also deduced that the *A. venetum* genome is complex. In the future, the ‘2+3’ (Illumina+PacBio) sequencing technique combination strategy should be employed to supplement the Hi-C technique and resequencing technique for whole-genome research in *A. venetum* and the analysis of gene differences between *A. venetum* from different sources. This can be used to identify genetic variation information, and genome assembly and annotation can be used to analyze key genes in *A. venetum* for fiber synthesis.

## Abbreviations

C value: Carbon value
Hi-C: High-through chromosome conformation capture
K-mer: A sequence of k characters in a string
MISA: MicroSatellite identification tool
NS: Nonstructural protein
SSR: simple sequence repeat
